# Mean Brixia Severity Scores among Symptomatic COVID-19 Patients in a Tertiary Care Centre: A Descriptive Cross-sectional Study

**DOI:** 10.31729/jnma.8165

**Published:** 2023-05-31

**Authors:** Amit Shrestha, Subodh Shrestha, Abhushan Siddhi Tuladhar, Rumita Kayastha, Roja Khanal, Ashlesha Chaudhary, Anish Bhattarai, Sneha Bimali

**Affiliations:** 1 Department of Radiology, Nepal Medical College and Teaching Hospital, Jorpati, Kathmandu, Nepal; 2 Department of Radiology, Om Hospital and Research Centre, Chabahil, Kathmandu, Nepal; 3 Department of Radiology, Madhyapur Thimi Hospital, Bhaktapur, Nepal; 4 Nepal Medical College and Teaching Hospital, Jorpati, Kathmandu, Nepal

**Keywords:** *COVID-19*, *Nepal*, *pneumonia*, *prevalence*, *x-ray*

## Abstract

**Introduction:**

COVID-19 is a highly contagious viral disease which escalated into a global pandemic since its outbreak on 31 December 2019. Chest X-rays are the most common investigation in suspected cases to diagnose and manage pneumonia. The aim of this study was to find out the mean Brixia severity scores among symptomatic COVID-19 patients in a tertiary care centre.

**Methods:**

A descriptive cross-sectional study was conducted among the chest X-rays of symptomatic COVID-19-positive patients of a tertiary care centre. Data from 1 May 2021 to 31 July 2021 were collected between 1 August 2022 and 1 January 2023 from the hospital records. Ethical approval was taken from Institutional Review Committee (Reference number: 01-079/080). Patients with reverse transcriptase polymerase chain reaction with symptoms of COVID-19 were included in this study. Convenience sampling method was used. Point estimate and 95% Confidence Interval were calculated.

**Results:**

Among the total of 300 patients, the mean Brixia severity score was 7.15±5.07 and out of 235 patients with abnormal chest X-ray, the mean Brixia severity score was 9.13±3.84. A total of 68 (22.66%) patients had mild, 115 (38.33%) had moderate and 52 (17.33%) had severe scores.

**Conclusions:**

The mean Brixia severity score among symptomatic COVID-19 patients was found to be higher than the other studies done in similar settings.

## INTRODUCTION

World Health Organization (WHO) announced an outbreak of multiple cases of pneumonia of unknown aetiology in Wuhan City, China on December 31, 2019.^1^ WHO named the disease "coronavirus disease 2019 (COVID-19)".^2^ It has since escalated into a global pandemic causing loss of millions of lives, morbidities and devastating socioeconomic impact. It causes severe inflammation of the respiratory system and will remain an important differential diagnosis for the foreseeable future.^3^

Chest X-rays (CxR) remain the most commonly performed investigation in suspected COVID-19 cases due to their low cost, easy availability and portability.^4,5^ There is only one published study on CxR findings of COVID-19 in Nepal.^6^ Typical abnormal chest X-ray findings in COVID-19 include bilateral peripheral mid and lower zones ground glass opacities (GGO) and/or consolidation/air space opacities (ASO) with pleural effusion being uncommon.^5,7^

The aim of this study was to find out the mean Brixia severity scores among symptomatic COVID-19 patients in a tertiary care centre.

## METHODS

This descriptive cross-sectional study was carried out on symptomatic RT-PCR-positive COVID-19 patients admitted to or visiting Nepal Medical College and Teaching Hospital, Jorpati, Kathmandu, Nepal. Data from 1 May 2021 to 31 July 2021 were collected between 1 August 2022 and 1 January 2023 from the hospital records. Ethical approval was taken from Institutional Review Committee of the same institute (Reference number: 01-079/080). Those patients with both RT-PCR positive and showing symptoms of COVID-19, like fever, cough, chest pain, myalgia, fatigue, shortness of breath, anosmia and ageusia were included in this study. Those patients with PCR positive without symptoms, follow-up cases of previously positive cases that were now negative and previous history of other chronic respiratory illnesses were excluded from this study. Convenience sampling method was used. The sample size was calculated using the following formula:


$$\begin{array}{ll} \text{n} & =\frac{{\text{Z}}^{2}\times \sigma^{2}}{{\text{e}}^{\text{2}}}\\ & =\frac{1.96^{2}\times 5.70^{2}}{0.70^{2}}\\ & =256 \end{array}$$

Where,

n = minimum required sample size
Z = 1.96 at 95% Confidence Interval (CI)
p = standard deviation taken as 5.7 from published literature^8^
q = 1-p
e = margin of error, 0.7

Here, the minimum required sample size was 256. However, a sample size of 300 was used for our study.

Radiological findings were described according to the Fleischner Society glossary of terms for thoracic imaging.^9^ GGO was defined as increased opacification of lung parenchyma not obscuring blood vessels and bronchi. ASO was described as homogenous opacification of lung parenchyma obscuring blood vessels and bronchi. The radiographic findings were classified as regards peripheral or central predominance (defined as halfway between the lateral edge of the lung and hilum), or neither; right, left, or bilateral lung affection and upper or lower zonal or no zonal predominance.^10^ CxR was evaluated for predominant pattern, selecting from GGO, ASO, or both, central or peripheral opacities; if none of these patterns applied the reviewers would select 'normal'.

The reviewer rated the severity score for the chest X-ray findings as normal, mild, moderate, or severe. Severity was based on the Brixia severity score system which divides the lungs into six zones on frontal chest projection: (i) upper zones: above the inferior wall of the aortic arch, (ii) middle zones: below the inferior wall of the aortic arch and above the inferior wall of the right inferior pulmonary vein (i.e. hilar structures), and (iii) lower zones: below the inferior wall of the right inferior pulmonary vein (i.e., lung bases).^11^ A score (from 0 to 3) is given to each zone based on the lung abnormalities detected on frontal chest projection, as follows: (i) score 0: no lung abnormalities, (ii) score 1: ground glass opacities, (iii) score 2: ground glass and airspace opacities(interstitial predominance), and (iv) score 3: ground glass and airspace opacities(alveolar predominance). The score of the 6 lung zones is summed to have an overall severity score ranging from 0 to 18. For ease of comparison, our patients were classified according to their total radiographic score into 4 groups; as follows: (i) normal: 0, (ii) mild group: from 1 to 6, (iii) moderate group: from 7 to 12, and (iv) severe group: from 13 to 18.^12^ Other chest X-ray findings such as pleural effusion, cardiomegaly, pulmonary vessel enlargement, and adenopathy were not included in the scoring system.

Data was entered and analyzed using IBM SPSS version 20. Point estimate and 95% CI was calculated.

## RESULTS

Among the total of 300 patients, the mean Brixia severity score was 7.15±5.07 and out of 235 patients with abnormal chest x-ray, the mean Brixia severity score was 9.13±3.84. Among them, the youngest was 5 years and the oldest was 99 years old with a mean age of 57.41 years (Table 1).

**Table 1:** Grading of patients with abnormal CxR (n= 300).

Severity	Mean age (years)	n (%)
Normal	45.10	68 (22.66)
Mild	55.03	68 (22.66)
Moderate	63.23	115 (38.33)
Severe	63.27	52 (17.33)

When the pattern of lung involvement was considered, out of a total of 1410 zones in 235 patients with abnormal CxRs (six zones in each patient), 1034 zones (73.33%) had abnormal opacities. Among abnormal opacities, 254 zones (24.56%) had GGO, 453 zones (43.81%) had mixed but predominant GGO and 327 (31.62%) had mixed but predominant ASO (Table 2).

**Table 2:** Pattern of lung involvement (n= 1034 zones).

Pattern	Number of zones n (%)
GGO	254 (24.56)
Mixed with GGO predominant	453 (43.81)
Mixed with ASO predominant	327 (31.62)

Peripheral distribution of opacities were seen in 657 zones (63.53%), central in eight zones (0.77%) and both central and peripheral in 356 zones (34.42%) (Table 3).

**Table 3:** Distribution of lung opacities (n= 1034).

Distribution	Number of zones n (%)
Peripheral	657 (63.53)
Central	8 (0.77)
Both	356 (34.42)

Out of 470 upper zones in 235 abnormal CxRs (two in each patient x 235) unilateral or bilateral abnormalities were seen in 212 zones (45.10%). Similarly out of 470 middle zones unilateral or bilateral abnormalities were seen in 432 zones (92.97%) and out of 470 lower zones unilateral or bilateral abnormalities were seen in 390 zones (82.97%) (Table 4).

**Table 4:** Zonal distribution of lung opacities (n= 470).

Distribution	Number of zones n (%)
Upper zone	212 (45.10)
Mid zone	432 (92.97)
Lower zone	390 (82.97)

Diffuse lung involvement was seen in 45 (19.14%) patients. Asymmetric cases including only mid-zone and lower-zone involvement were seen in 13 (5.53%) cases.

Out of 235 abnormal CxRs, 218 (92.76%) had bilateral and 17 (7.34%) had unilateral disease. Out of 17 unilateral cases, mid-zone involvement was seen in all 17 cases (100%), and upper-zone involvement was noted in only 4 cases (23.52%) and was always associated with involvement of mid and lower zones. Lower zone involvement was seen in 13 cases (76.47%). Similarly, among unilateral cases, ground glass opacities were seen in 5 (29.41%), mixed opacities with ground glass predominance in 10 (58.82%), and with air space opacities predominance in only two patients (11.76%). Total 16 (94.11%) had mild, 1 case (5.88%) had moderate severity out of 17 unilateral cases and none had severe disease.

## DISCUSSION

In our study, among the total of 300 patients, the mean Brixia severity score was 7.15±5.07 and out of 235 patients with abnormal chest x-ray, the mean Brixia severity score was 9.13±3.84. Whereas in one of the study, the mean score was found to be 6.9±5.7 which is lower than our finding.^8^ In our study, only 52 (22.12%) out of 235 positive CxRs showed classic abnormalities of bilateral mid and lower zone peripheral grade 1, 2, or 3 opacities without pleural effusion whereas CXR findings in COVID-19 are described in the literature, but the data are relatively scant as compared to CT. A sole study of CxR in COVID-19 conducted in Nepal as early as May 2021, showed that among 111 RT-PCR positive cases, six patients (5.4%) had normal chest X-rays, classic/probable COVID-19 picture was present in 79 (71.1%) patients while eight (7.2%) had intermediate for COVID-19 X-ray findings.^6^

Among 79 patients with classic/probable COVID-19 CXR findings, 71 (89.8%) had bilateral consolidation/ground glass opacities, 72 (91.1%) had peripheral lung involvement and 66 (83.5%) had middle and lower zone involvement. This study was based on the British society of thoracic imaging (BSTI) COVID-19 CxR classification whose definition of classic/probable disease was not exclusive for bilateral disease. This may be the reason our study showed only 22.12% of abnormal CxRs to have a classic appearance compared to 71.1% in the above study because our study limited classic appearances exclusively to bilateral opacities and to the absence of pleural effusion.^6^ Moreover, upper zone involvement was excluded from the classic appearance in our study. Our study showed a comparable frequency of bilateral (92.7% compared to 89.8%) and unilateral (7.3% compared to 7.2%) disease. However, 22.2% of our patients showed diffuse bilateral lung involvement compared to 10.1% in theirs.^6^ When comparing distribution, peripheral opacities were seen in 63.6% and both peripheral and central opacities in 35.6% of our patients compared to 91.1% and 10.0% respectively in theirs.^6^ Mid and lower-zone involvement was seen in 83.5% and midzone in only 16.5% in the above study whereas our study showed 72.0% had mid and 65.0% had lower zone involvement.^6^

Also commonly noted were GGO 33%, peripheral infiltrates 41%, lower lung zone involvement 50% and bilaterality 50% compared to 57.4%, 63.6%, 65%, and 72% respectively in our study. Hence, bilateral peripheral lower zone GGO was more frequent in our study. They found peak severity on CXR at 10-12 days from the onset of symptoms. They concluded that CXR depicts similar findings as CT.

Similarly, a recent study out of Italy retrospectively analyzed 240 RT-PCR-positive cases to determine the most common lung alterations found using CXR with respect to time since symptom onset.^13^ Abnormalities most frequently occurred bilaterally and peripherally, with reticular alteration being the most common finding in the early phases of the disease. GGO became predominant in later phases and consolidation while occurring less frequently, and also increased with time. CXR showed abnormalities in 75.0% of the RT-PCR confirmed cases compared to 78.3% in our study. Therefore, while it has not generally been recommended due to low sensitivity, these observations confirm recent suggestions that CXR should be considered a feasible imaging technique in diagnosing COVID pneumonia.

A study in Pakistan showed an increased incidence of bilateral middle zone involvement.^14^ This was similar to our study which showed mid zone (72.0%) to be most frequently involved compared to lower (65.0%) and upper (35.3%) zones.

Our study correlated best with the single largest study on CxR of COVID patients to date. This was a multicentric cross-sectional study conducted in 2485 RT-PCR-confirmed COVID-19 patients during the peak of the 2020 pandemic wave in Qatar.^7^ It showed the prevalence of abnormal findings in only 16.7% of cases with GGO in 386 (92.6%), consolidation in 95 (22.8%) and pleural effusion in 10 (2.4%) patients. Upperzone involvement was seen in 44 (10.6%), mid-zone in 200 (48.0%) and lower zone in 363 (87.1%) patients. Peripheral distribution was seen in 290 (69.5%), central in 16 (3.8%) and both in 107 (25.7%) patients. Unlike our study which showed abnormal CxR in 78.3%, the above study found CxR to have only 16.7% sensitivity because they included both symptomatic and asymptomatic patients and studies have shown that subclinical COVID-19 infection has a prevalence as high as 40 to 45%.^15^

Also, age is another factor which determines the prevalence of clinical and subclinical infection as shown by studies where clinical symptoms manifested in 21% of infections in ten to 19-year-olds, rising to 69% in people aged over 70 years.^16^ This indicates that the older age group has more likelihood of developing symptoms than the younger population. Our study showed more frequent involvement of mid zones (72.0% compared to 48.0%) and lesser in lower zones (65% compared to 87.1%).

In a study done in Jordan during the first outbreak in March 2020, a total of 190 chest X-rays were obtained from 88 patients admitted to the hospital with confirmed COVID-19 and of 45% of symptomatic patients, only 14.8% showed abnormal chest X-ray findings.^17^ The most common finding on chest X-rays was peripheral GGO affecting the lower lobes. In the course of illness, the GGO progressed into consolidations peaking around 6-11 days (GGO 70%, consolidations 30%). The consolidations regressed into GGO towards the later phase of the illness at 1217 days (GGO 80%, consolidations 10%). There was an increase in the frequency of normal chest X-rays from 9% on the 6th to the 11^th^ day up to 33% after 18 days indicating a healing phase.

Our study was cross-sectional point study and did not evaluate temporal changes of chest x-ray opacities in the same patient.

## CONCLUSIONS

The mean Brixia severity score among symptomatic COVID-19 patients was found to be higher than the other studies done in similar settings.
